# Wandering starch – how plastid dynamics affect starch granule formation?

**DOI:** 10.1111/nph.70925

**Published:** 2026-01-16

**Authors:** James R. Lloyd

**Affiliations:** ^1^ Department of Genetics, Institute for Plant Breeding and Biotechnology Stellenbosch University Private Bag X1, Stellenbosch 7599 South Africa

**Keywords:** amyloplast movement, anisotropic growth, cereal endosperm, granule movement, simple starch granules, starch granule initiation, starch granule shape, stromule

## Abstract

This article is a Commentary on Esch *et al*. (2026), **249**: 2877–2893.

Starch is the major carbon store of most plants and is widely used in many industrial processes (Zeeman *et al*., 2010). It is synthesized in plastids, which can be chloroplasts in photosynthetic tissue or amyloplasts in starch storing storage organs. Starch granule size can affect its industrial usability, and different plants accumulate different patterns of starch granule shapes within their amyloplasts. In cereal endosperm, three patterns of granule formations occur (Fig. 1). Some (such as *Brachypodium distachyon*) contain simple starch granules (Tanackovic *et al*., 2014), which have been hypothesized to develop from a single initiation event per amyloplast. Others (such as rice) develop compound granules in which multiple granules are initiated within amyloplasts and increase in size as the endosperm develops, eventually impacting each other due to space constraints (Matsushima *et al*., 2015). This leads to the formation of an angular tessellated compound granule composed of all these initiation events. Finally, some cereals (such as barley) accumulate bimodal granules in which two waves of starch granule initiation events occur at different points during endosperm development, leading to the formation of distinct large and small granule populations. The first wave leads to the formation of large granules, and the later wave to small granules (Matsushima & Hisano, 2019; Kamble *et al*., 2023). A paper published in this issue of *New Phytologist* (Esch *et al*., 2026; pp. 2877–2893) examines the formation of simple starch granules in *B. distachyon*. Contrary to the hypothesis that they are initiated from one event per amyloplast, they demonstrate that these granules can arise from multiple initiations within amyloplasts and provide evidence as to why this does not lead to the formation of compound granules. Interestingly, they hypothesize that the granules remain separated due to their ability to move within an amyloplast.

*It is the first study, which suggests that plastid dynamics might affect starch granule shape, something that had previously been assumed to be solely determined by catalytic and noncatalytic proteins …*

**Fig. 1:**
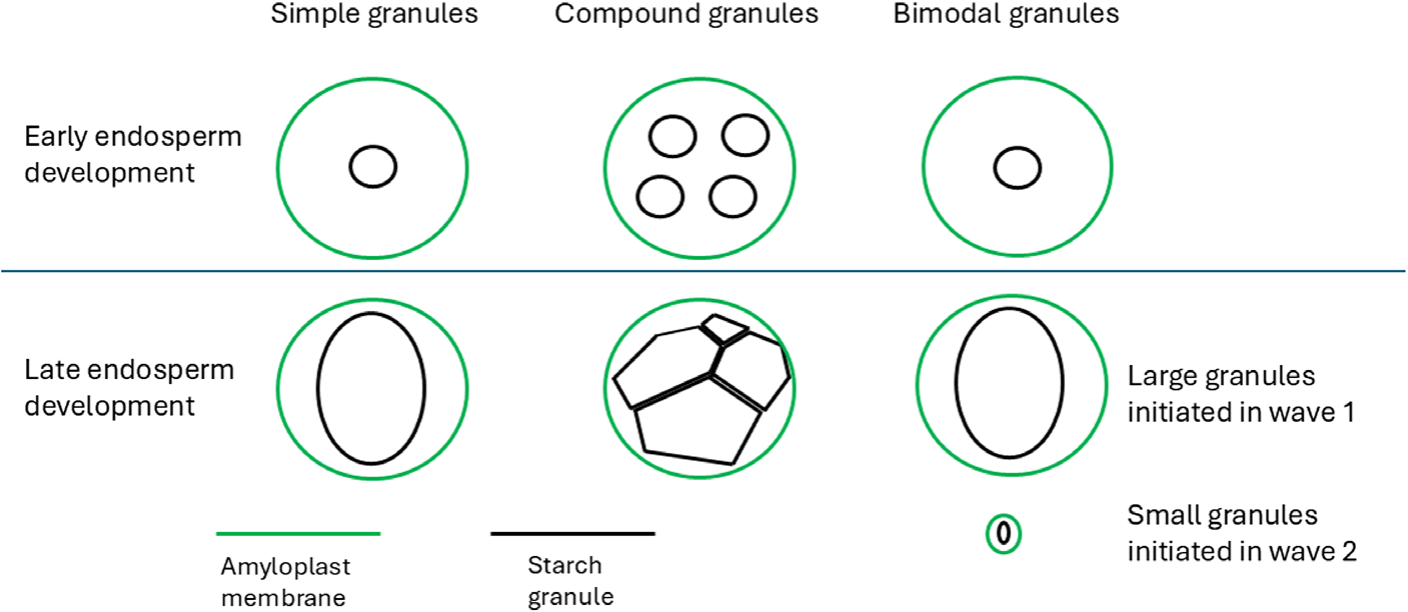
Three types of starch granule accumulation occur in cereal endosperm. Some species accumulate simple starch granules in which each granule results from one initiation event early in endosperm development. Other species accumulate compound granules in which multiple granules are formed in amyloplasts and impact each other during endosperm development, leading to an angular appearance. Finally, some species synthesis bimodal granules in which two waves of granule initiation events occur. The first wave occurs early in development and leads to the synthesis of large granules, and the second occurs later in development, leading to small granules.

Starch is a glucose biopolymer composed of α1,4 chains linked together by α1,6 branchpoints. Granules consist of two structurally related, but distinct polymers, amylose and amylopectin. Amylose contains chains of several hundred glucose moieties linked by α1,4 bonds, with occasional α1,6 branchpoints, whilst amylopectin contains many shorter (6–50 glucose moieties) α1,4 chains linked by branchpoints and help form a highly ordered semicrystalline structure (Zeeman *et al*., 2010). The α1,4 chains are synthesized by starch synthase enzymes using ADP‐glucose, whilst the branchpoints are introduced by starch branching enzymes. All angiosperms contain multiple isoforms of these enzymes, which often demonstrate different activities in terms of the average length of the glucan chain they synthesize or the frequency of the branchpoints they introduce. They work together to synthesize the complex starch granule structure, and it is known that starch granule size and shape can be altered through mutating starch synthases or starch branching enzymes (Seung & Smith, 2019; Mérida & Fettke, 2021). The alterations in shape are most likely due to changes in the ratio of amylose to amylopectin, or the branching structure of amylopectin within starch caused by the absence of the mutated enzyme.

A number of proteins that can act to synthesize or degrade starch have been demonstrated to be involved in starch granule initiation in the cereal endosperm (Seung & Smith, 2019; Hawkins *et al*., 2021; Kamble *et al*., 2023). Some noncatalytic proteins have also been implicated in starch granule initiation in other species, including PROTEIN TARGETING TO STARCH, PROTEIN INVOLVED IN STARCH INITIATION I, STARCH SYNTHASE V and MYOSIN RESEMBLING CHLOROPLAST PROTEIN (Seung & Smith, 2019; Mérida & Fettke, 2021). The plastid itself has often not been considered as being involved in this, despite multiple strands of evidence demonstrating that its structure can affect starch granule size and shape. These include analysis of mutants affecting amyloplast membrane properties or amyloplast shape and size, which affect granule morphology (Myers *et al*., 2011; Wang *et al*., 2020; Esch *et al*., 2023). Few studies have examined whether plastid dynamics are involved in altering starch granules, mainly because of the technical difficulty in doing so. The study of Esch *et al*. (2026) overcomes some of these difficulties by targeting mCHERRY to the stroma of *B. distachyon* plastids, allowing easy visualization of amyloplasts both in live cell imaging and after isolation.

Using these transgenic plants, they examined the number of granules at four time points. Although the number of starch granules increased between the two earliest sampling points, they remained stable after that, indicating that all granule initiation events were complete by the second sampling time. Interestingly, many amyloplasts contained more than one granule at all sampling times, demonstrating that the presence of multiple granule initiation events within amyloplasts occurs at an early stage (Fig. 2). This observation raises the question as to why these do not form compound granules at maturity. One possibility would be that internal membranes could physically separate the granules, but these were never observed in their experiments. To examine this further, the authors used live cell imaging of immature cells to examine amyloplasts and identified several features. First, the shape of amyloplasts was highly variable, often being similar in shape to the starch granule within them. These morphological features were retained after the isolation of the amyloplasts, which raises the intriguing possibility that the shapes are formed by intrinsic plastid mechanisms, rather than interactions with, for example, the cytoskeleton. Second, amyloplasts are connected by long‐lived membrane bound stromules, which are likely to allow the movement of metabolites between amyloplasts (Fig. 2). They also noticed small, short‐lived stromules, which form transiently from the plastid membrane and the function of which is unclear. Finally, they demonstrate that amyloplasts move within the cell. Using an actin polymerization inhibitor, they demonstrated that this movement is reliant on cytoplasmic streaming.

**Fig. 2:**
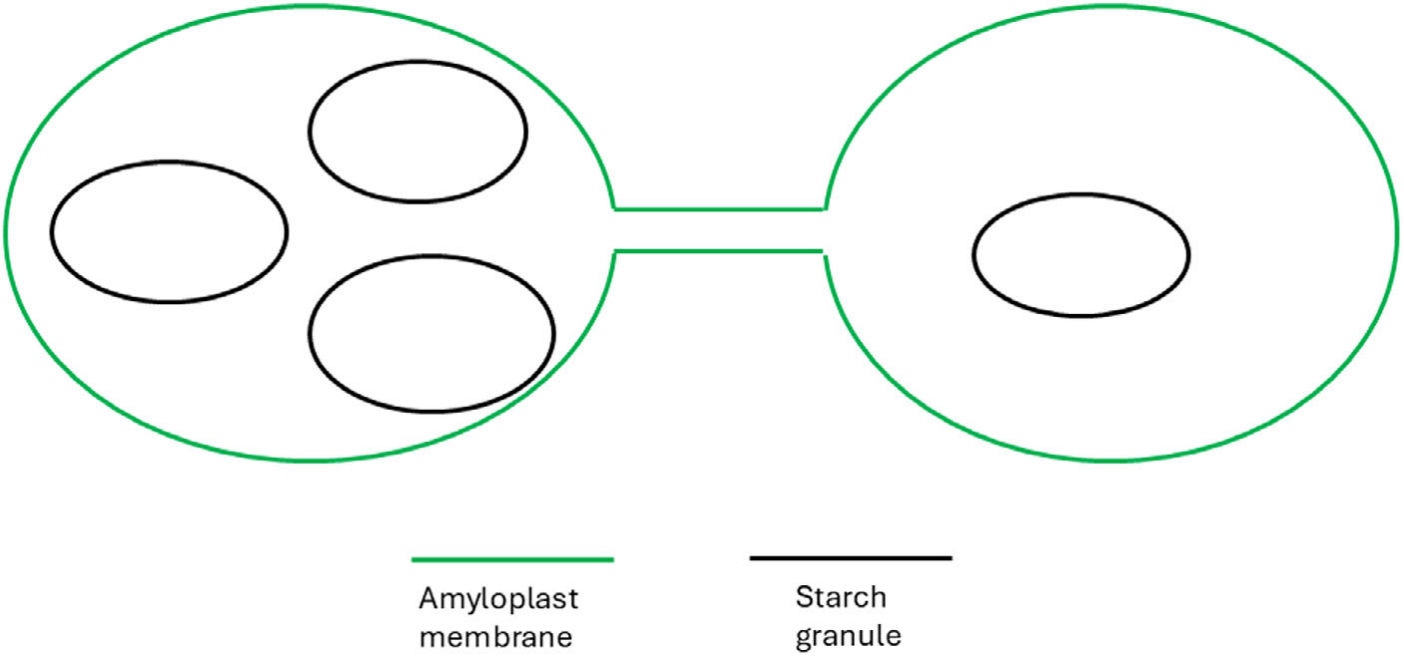
Starch synthesis in *Brachypodium distachyon*. Amyloplasts are joined by long‐lived stromules and can contain one, or small numbers of, simple starch granules. It is possible for amyloplasts to become stretched, leading to anisotropic starch granule growth and the development of oval‐shaped granules.

Interestingly, the authors noticed that not only do amyloplasts move within the immature endosperm cell but starch granules also move within the amyloplast. They propose that this movement is a mechanism that allows multiple starch granules present within an individual amyloplast to avoid contact with each other whilst they grow which allows them to remain as simple granules. The authors also propose that plastid dynamics may affect starch granule shape. *Brachypodium* starch granules are ovoid, indicating that they grow anisotropically. A Solanaceae specific isoform of PROTEIN TARGETING TO STARCH is known to regulate anisotropic granule growth in potato tubers (Hochmuth *et al*., 2025), but as this isoform is absent in cereals, it cannot be involved. Other proteins may be regulating this, but Esch *et al*. (2026) suggest that amyloplasts connected by stromules would create membrane tension along one axis that influence granule shape.

This study is significant for several reasons, but it also raises questions that remain to be answered. It is the first study, which suggests that plastid dynamics might affect starch granule shape, something that had previously been assumed to be solely determined by catalytic and noncatalytic proteins involved in synthesizing starch and/or initiating granule formation. As the study was performed in cereal endosperm, the question arises as to whether this is also true in other plant species and other tissue types. The presence of stromules linking plastids has been observed previously (Hanson & Hines, 2018), but it is unclear how they form and what their significance in plastid biology is. The role of the short‐lived stromules is also unclear. It has been observed in cereals exhibiting bimodal starch granule accumulation that some of the small granules initiate within these stromules (Kamble *et al*., 2023) but that is not the case in *Brachypodium*, in which all granules are initiated earlier in endosperm development. Finally, what is the significance of granule movement within amyloplasts, and how does it occur? *Brachypodium* endosperm accumulates less starch than cereals that have undergone more intensive breeding efforts, and so it could be argued that the simple granules can remain apart from each other in *Brachypodium* amyloplasts as there is simply less starch. It would be useful to try the techniques used in this study to examine starch synthesis within cereals, such as maize, which accumulate more endosperm starch than *Brachypodium*, but still develop simple starch granules.

Although this study has concentrated on starch granule formation, the type of technology that it uses will also help in the study of plastids more generally. This critical organelle contains many essential pathways, but the role of plastid dynamics in their function is only starting to be examined. Gaining a better understanding of this will allow plastid improvement in ways that might increase plant productivity, and this type of technology is an important step in their study.

## Disclaimer

The New Phytologist Foundation remains neutral with regard to jurisdictional claims in maps and in any institutional affiliations.
